# Effect of Lemon Waste Natural Dye and Essential Oil Loaded into Laminar Nanoclays on Thermomechanical and Color Properties of Polyester Based Bionanocomposites

**DOI:** 10.3390/polym12071451

**Published:** 2020-06-28

**Authors:** Bàrbara Micó-Vicent, Valentin Viqueira, Marina Ramos, Francesca Luzi, Franco Dominici, Luigi Torre, Alfonso Jiménez, Debora Puglia, María Carmen Garrigós

**Affiliations:** 1 Colour and Vision Group, University of Alicante, San Vicente del Raspeig, ES-03690 Alicante, Spain; barbara.mico@ua.es (B.M.-V.); valentin.viqueira@ua.es (V.V.); 2 Department of Analytical Chemistry, Nutrition & Food Sciences, University of Alicante, San Vicente del Raspeig, ES-03690 Alicante, Spain; marina.ramos@ua.es (M.R.); alfjimenez@ua.es (A.J.); 3 Department of Civil and Environmental Engineering, University of Perugia, 05100 Terni, Italy; francesca.luzi@unipg.it (F.L.); francodominici1@gmail.com (F.D.); luigi.torre@unipg.it (L.T.); 4 Department of Appl. Stat. & Operat. Research, & Qual., Universitat Politècnica de València, ES-03801 Valencia, Spain

**Keywords:** lemon waste, natural pigments, essential oils, experimental design, nanoclays, bionanocomposites

## Abstract

In this work, polyester-based nanocomposites added with laminar nanoclays (calcined hydrotalcite, HT, and montmorillonite, MMT) loaded with lemon waste natural dye (LD) and essential oil (LEO) were prepared and characterized. The optimal conditions to synthetize the hybrid materials were obtained by using statistically designed experiments. The maximum LD adsorption with HT was found using 5 wt% of surfactant (sodium dodecyl sulfate), 5 wt% of mordant (aluminum potassium sulfate dodecahydrate) and 50% (*v/v*) ethanol. For MMT, 10 wt% of surfactant (cetylpyridinium bromide), 5 wt% of mordant, 1 wt% of (3-aminopropyl) triethoxysilane and 100% distilled water were used. LEO adsorption at 300 wt% was maximized with MMT, 10 wt% of surfactant and 50 °C following an evaporation/adsorption process. The obtained hybrid nanofillers were incorporated in a polyester-based matrix (INZEA) at different loadings (3, 5, and 7 wt%) and the obtained samples were characterized in terms of thermal stability, tensile behavior, and color properties. HT_LEM-based samples showed a bright yellow color compared to MMT_LEM ones. The presence of lemon hybrid pigments in INZEA-based systems produced a remarkable variation in CIELAB color space values, which was more visible with increasing the nanofillers ratio. A limited mechanical enhancement and reduced thermal stability was observed with the nanopigments addition, suggesting a limited extent of intercalation/exfoliation of MMT and HT in the polymer matrix. MMT_LEM pigments showed higher thermal stability than HT_LEM ones. A significant increase in Young’s modulus of nanocomposites loaded with hybrid LEO was observed compared to the biopolymer matrix. The LEO inclusion into the nanoclays efficiently improved its thermal stability, especially for MMT.

## 1. Introduction

Ecological concerns related to the use of synthetic dyes have motivated industries to consider the replacement of these compounds by eco-friendly non-toxic natural dyes to minimize the negative environmental impact and health hazardous problems related to cytotoxicity, genotoxicity, and carcinogenicity of synthetic dyes [1,2,3]. Natural dyes and pigments have been also used as polymer additives due to their antimicrobial, antioxidant or deodorant properties [4].

Natural dyes can be extracted from natural sources such as food waste [5]. In particular, lemon waste could be a potential source for the extraction of natural yellow dyes for the dyeing industry [6]. Extracts from lemon sources have been widely studied because of their medicinal properties, showing their main components such as citric acid, ascorbic acid, minerals, and flavonoids, antioxidative, anti-inflammatory, antiallergic, antiviral, antiproliferative, antimutagenic, and anticarcinogenic activities [7]. Lemon dyes are widely used for coloring and medicine [8]. However, natural dyes could present several drawbacks such as low light fastness, difficult color reproduction between different natural dye samples, limited color range, poor color fastness and reduced chemical and thermal stability [4,9,10]. Regarding essential oils (EOs), they have been also considered as functional additives in food packaging and cosmetic sectors due to their antibiotic and flavoring properties [11]. However, these compounds are highly volatile and biodegradable showing low persistency in the environment [12]. These drawbacks have limited natural dyes and EOs to be used in industrial applications.

Nanoclays, such as laminar montmorillonite (MMT), or layered double hydroxides as hydrotalcite (HT), have been used as hosts for organic or natural dyes. It has been demonstrated that thermal stability and color fastness can be improved by the hybridization of pigments with different nanoclay structures [13,14,15,16]. Depending on the nanoclay exchange capacity, some surface modifications should be needed, such as the use of surfactants [17,18,19] or silane couple agents to open the nanoclays structure and improve their adsorption capacity. Additionally, it has been proved that other additives used with natural dyes, such as mordant salts, can improve the natural dye–nanoclay interactions [4]. In another approach, Guillermin et al. [20] developed hybrid materials based on montmorillonite, polydiallyldimethylammonium chloride, as cationic polymer, and carminic acid. The presence of the cationic polymer modified the surface charge of montmorillonite favoring the carminic acid adsorption.

Similarly, organo-modified nanoclay essential oil hybrids have been used to improve EOs stability for different applications, such as pest repellents, anti-inflammatories and antioxidants [21,22]. The high volatility of EOs is their main drawback in several applications that require temperature processing, such as in the case of polymer nanocomposites, leading to EO losses by evaporation during polymer processing [23]. The encapsulation of EOs into nanoclays has been proved to be suitable for protecting and preserving the effectiveness of the EO bioactive substances in storage, providing a controlled release of EOs into the polymer matrix [24]. The preparation of MMT-essential oil powders with oregano, thyme and basil oils was reported showing a significant modification in the basal space of MMT clay and increased EO thermal stability with the EO adsorption [17,25]. Nanomaterials with different EO components were prepared by Bernardos et al. [26] by adsorption onto a montmorillonite nanoclay, obtaining enhanced antifungal activity against *Aspergillus niger* and *Staphylococcus aureus* compared to the corresponding free forms. The biocidal efficiency of alginate-MMT/lemon EO films against two fungal and four bacterial strains was addressed by Hammoudi et al. [27] for the potential use in antimicrobial food packaging applications.

It is well known that, in general, mechanical and thermal properties of polymer matrices can be improved by using exfoliated nanoclays (organomodified with surfactant or silane components) [28,29,30,31,32,33,34,35]. Falling in this category, nanoclay-based pigments or nanopigments have been applied mainly for coloring polymers, acting as reinforcing fillers and colorants at the same time. Many studies have been carried out to assess the influence of nanopigments in thermoplastic polymers. Marchante et al. [36] prepared and characterized a series of dye-clay nanopigments and studied their effects in linear low-density polyethylene (LDPE) and ethylene vinyl acetate (EVA) composites. They found that polymer composites containing the organic-inorganic pigments exhibited better color characteristics and improved mechanical properties compared to composites with conventional pigments. Beetroot red extract, carotene and copper chlorophyll were used to obtain MMT and HT-based hybrid natural pigments which were incorporated in an epoxy bioresin. Different modifiers (surfactant, coupling agent (silane) and a mordant salt (alum)) were combined to improve the natural dyes adsorption. The results demonstrated an improvement in thermal stability, color performance and UV-VIS light exposure stability for the obtained bionanocomposites [37].

The aim of this work is the incorporation of lemon dye and EO, both extracted from agricultural wastes, into different nanoclay structures to obtain hybrid natural EO and dye nanomaterials to be used as additives in biopolymer matrices. For this purpose, statistical experimental designs were used for the optimization of the hybrid pigments and EO-nanoclays preparation, followed by the development of polyester-based nanocomposites at different additives loading (3, 5 and 7 wt%) which were characterized on their thermal, mechanical and color properties.

## 2. Materials and Methods

### 2.1. Materials and Reagents

Lemon waste was obtained from discarded whole fruits from FECOAM (Murcia, Spain). The flavedo was mechanically removed and the obtained peel was slightly cut into small pieces. A household blade cutter was used for 10–20 s at medium speed to reach particles of 1–50 mm^3^.

Montmorillonite (MMT, Gel White) and hydrotalcite (HT, BioUltra, ≥99.0%) laminar nanoclays, with a different charge ion capacities, were supplied by Southern Clay Products (Gonzales, TX, USA) and Sigma-Aldrich (St. Louis, MO, USA), respectively. HT was calcined at 600 °C for 3 h before use. Cetylpyridinium bromide (CPB) and sodium dodecyl sulfate (SDS) were used as surfactants for MMT and HT, respectively. A mordant salt, aluminum potassium sulfate dodecahydrate, and a coupling agent, (3-Aminopropyl) triethoxysilane, were also used as modifiers. All of these reagents and other chemicals were of analytical grade and they were purchased from Sigma-Aldrich.

For bionanocomposites preparation, INZEAF2 biopolyester commercial grade (density of 1.23 g cm^−3^ measured at 23 °C, moisture content <0.5%, melt flow rate of 19 g/10 min (2.16 kg, 190 °C)) was kindly supplied by Nurel (Zaragoza, Spain).

### 2.2. Lemon Waste Dye and Essential Oil Extraction

A FLEXIWAVE™ microwave oven (Milestone srl, Bergamo, Italy) was used to obtain natural lemon dye (LD) and lemon essential oil (LEO) by following a method previously optimized. For LEO, microwave-assisted hydrodistillation (MAHD) was used. An amount of 750 g of solid waste was used for extraction with a water to solid ratio of 0.3. The MAHD process was divided in a first step with sample heated near to the boiling temperature (applying 1.2 W g^−1^ for 5 min) and a second step where the oil was distilled applying 0.7 W g^−1^ for 10 min. The collected oil phase was centrifuged at 4000 G for 10 min to separate water and oil phases getting a transparent EO extract.

After LEO extraction, the remaining sample was used for subsequent LD microwave-assisted extraction (MAE). Of the remaining sample, 6 g was mixed with 80% (*v/v*) ethanol in water, at a liquid to solid ratio of 1:10, in a 250 mL round-bottom flask. Then, samples were heated in the microwave oven at 80 °C for 5 min at 500 W of microwave power. A heating rate and stirring rate of 20 °C min^−1^ and 400 rpm were used, respectively. The obtained extracts were filtered and polysaccharide compounds were precipitated by adding 96% (*v/v*) ethanol. Samples were kept overnight in a freezer at −20 °C to promote precipitation of insoluble compounds and they were vacuum-filtered. The ethanol present in the samples was then removed in a rotary evaporator (R-300, Büchi Labortechnik AG, Switzerland) and the aqueous solution was freeze-dried (LyoQuest Plus, Telstar, Terrassa, Spain). The purified samples were stored in the darkness at room temperature until further use.

### 2.3. Synthesis of Lemon-Hybrid Nanoclay Systems with Pigment and Essential Oil

#### 2.3.1. Lemon Hybrid Dye–Nanoclay Systems

For the synthesis of the lemon hybrid dye–nanoclay systems, the water/organic solvent dispersion method was followed, based on previous research [4,37]. The best synthesis conditions to maximize lemon dye adsorption were studied by using a 2^4−1^ fractional factorial design of experiments (DoE) using 50% (*v/v*) ethanol as solvent. Four independent variables at two levels were considered (Table 1): nanoclay ion exchange capacity, surfactant concentration, mordant concentration and silane (coupling agent) concentration. LD (2.2 g L^−1^) was used and the levels for the additive concentrations (Table 1**)** were selected to minimize the aggregation of particles since this is one of the main problems limiting the synthesis of hybrid pigments with MMT. The dye adsorbed over the initially added dye (%) was used as response to be maximized in the DoE analysis.

The main steps involved in the production of the yellow hybrid nanopigments mainly consisted of (1) the solubilization of the dye, dispersion of the nanoclay and exchange step; (2) a centrifugation and washing step to obtain the pigment paste; (3) a drying step by lyophilization to obtain a pigment powder; (4) a hand milling step (if needed) to avoid forming agglomerates. In the case of MMT, some particle agglomeration was observed making necessary to introduce an extra hand-milling step in the procedure to break the rock-like powder obtained for the production of the MMT-lemon hybrid pigments. According to this behavior, MMT-based experiments were replicated using 100% distilled water instead of 50% (*v/v*) ethanol as solvent (Table 2, coded as “w”), obtaining a fine dried powder pigment without the need of incorporating the fourth extra milling step. The determination of the adsorbed lemon dye in the hybrid nanoclay systems was determined using a UV–Vis spectrophotometer (JASCO V650, Easton, MD, USA) at 340 nm. Calibration curves were prepared in 50% (*v/v*) ethanol or water, depending on the synthesis conditions used (Table 2).

#### 2.3.2. Lemon Hybrid EO-Nanoclay Systems

The best synthesis conditions for lemon hybrid EO-nanoclay systems were studied by using a 2^1^•3^1^ fractional factorial DoE. The influence of the nanoclay type (HT or MMT) and different surfactant concentrations (1, 5, 10 wt%) on the LEO adsorption was evaluated (Table 3). A LEO content of 100 wt% (the same as nanoclay) was used. These values were selected according to previous literature dealing with the use of nanoclays and EOs adsorption [32]. Distilled water was used with MMT and 50% (*v/v*) ethanol was employed for HT, as solvents. The water/organic solvent dispersion method used for LD was followed also for LEO. A first mixing step of the surfactant and LEO was included according to Tornuk et al. [32], who developed LLDPE-based active nanocomposite films with nanoclays impregnated with essential oil constituents, such as thymol, eugenol and carvacrol, confirming the presence of the studied compounds after the development of the nanocomposites.

As a second approach, considering the previously obtained results, the synthesis process previously optimized for MMT with 10 wt% of CPB was modified by increasing LEO up to 300 wt% with the aim of maintaining the lemon presence in the final polymer samples. Three different experiments (Table 4) were carried out to evaluate the LEO adsorption on MMT nanoclay. The first sample EO.WO_300 was synthesized following the same conditions used for 100 wt% LEO and MMT by increasing LEO content up to 300 wt%. The synthesis was performed using a previous mixture of LEO and CPB, which were added to a water-MMT dispersion. After the exchange step, the solutions were centrifuged and the resulting sample was freeze-dried to obtain a fine powder. The second and third samples were synthetized using an evaporation/adsorption process according to the procedure reported by Giannakas et al. [17]. EO.WC.T50_300 was synthesized by using the same starting conditions previously described, but closing the dispersion recipient after the addition of the LEO–CPB mixture to MMT. Then, the temperature was increased until 50 °C under continuous stirring at 1500 rpm during 24 h. Finally, EO.WC.T100_300, MMT was synthetized by firstly modifying MMT with CPB using a water dispersion. Then, the solvent was separated by centrifugation obtaining a CPB–MMT paste which was mixed with 300 wt% LEO in a closed recipient and placed in an oven at 100 °C for 24 h. After separating the liquid from the mixture, the resulting paste was obtained by freeze-drying.

LEO adsorption was determined in the separated solvents after functionalization using a UV–Vis spectrophotometer (JASCO V650, Easton, MD, USA) at 220 nm and 265 nm, corresponding to the UV absorbances of limonene, main component present in LEO [38].

### 2.4. Bionanocomposites Preparation

Bionanocomposites based on INZEAF2 were obtained by melt blending the biopolymer and the synthetized lemon-hybrid additives (containing LD or LEO) at three different contents: 3, 5, and 7 wt%. A co-rotating twin-screw extruder, Xplore 5 and 15 Micro Compounder by DSM, was used by mixing at a rotating speed of 90 rpm for 3 min, setting a temperature profile of 190-195-200 °C in the three heating zones from feeding section to die. A Micro Injection Moulding Machine 10cc by DSM, coupled to the extruder and equipped with adequate molds, was used to produce samples for tensile tests according to the standards. An appropriate pressure/time profile was used for the injection of each type of samples, while the temperatures of the injection barrel and the molds were set, respectively, at 210 and 30 °C.

### 2.5. Bionanocomposites Characterization

Thermal degradation of lemon hybrid nanoclay systems and bio-nanocomposites was evaluated by thermogravimetric analysis (TGA, Seiko Exstar 6300, Tokyo, Japan). Around 5 mg of samples were heated from 30 to 600 °C at 10 °C min^−1^ under nitrogen atmosphere (200 mL min^−1^).

Differential scanning calorimetry (DSC) tests for bio-nanocomposites were conducted using a DSC Q1000 (TA Instruments, New castle, DE, USA) under nitrogen atmosphere (50 mL min^−1^). Samples of 3 mg were introduced in aluminum pans (40 µL) and they were submitted to the following thermal program: −30 °C to 250 °C at 10 °C min^−1^, with two heating and one cooling scans.

Tensile tests were carried out for bio-nanocomposites by using a universal test machine LK30 (Lloyd Instruments Ltd) at room temperature according to ASTM D638-14 Standard. A minimum of five different samples were tested using a 5 kN load cell, setting the crosshead speed to 5 mm min^−1^. Values for the different parameters (strength at break σ_b_, strain at break ε_b_ and Young Modulus, E) have been collected.

Optical properties of bio-nanocomposites were studied with a Konica Minolta sphere integrated spectrophotometer (CM-2300d, Tokyo, Japan). Data were acquired by using the SCI 10/D65 method whereas CIELAB color variables, as defined by the Commission Internationale de 1’Éclairage (CIE 1995), were used. Samples were placed on a white standard plate and L*, a*, and b* parameters were determined. L* value ranges from 0 (black) to 100 (white); a* value ranges from -60 (green) to 60 (red); and b* value ranges from −60 (blue) to 60 (yellow). Measurements were performed, in triplicate, at random locations on each sample. Total color difference ΔE_ab_* was calculated with the colorimetric attributes of the CIELAB color space.

### 2.6. Statistical Analysis

Statgraphics Centurion XVI (Statistical Graphics, Rockville, MD, USA) was used to generate and analyze the results of the experimental design. The graphic analysis of the main effects and interactions between variables was used and the analysis of variance (ANOVA) was carried out.

Statistical analysis of experimental data was performed by one-way analysis of variance (ANOVA) using SPSS 15.0 (Chicago, IL, USA) and expressed as means ± standard deviation. Differences between average values were assessed based on the Tukey test at a confidence level of 95% (*p* < 0.05).

## 3. Results

### 3.1. Lemon Hybrid Pigments

The optimal synthesis conditions of lemon hybrid pigments were obtained by calculating the dye adsorption as the concentration difference between the initially added dye in the nanoclay dispersion and the dye separated from the solvent after centrifugation. As shown in Table 2, the obtained synthesis performance was higher than 98% in all experiments, representing very good values and ensuring that mainly all the initial dye added to both nanoclay structures was successfully incorporated. Better adsorption results were found using MMT and 100% water compared to those obtained with MMT and 50% (*v/v*) ethanol as solvent.

**Figure 1:**
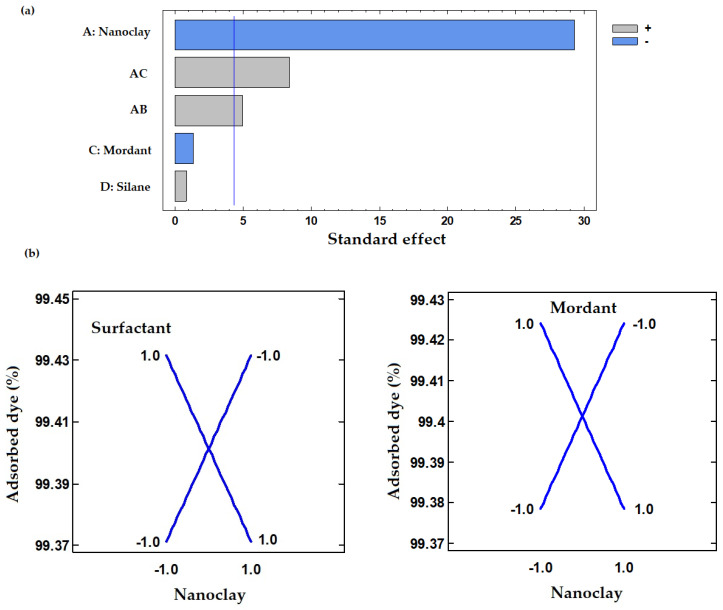
Pareto (**a**) and interactions (**b**) plots obtained to maximize the adsorption of lemon dye into the nanoclays.

Analysis of variance (ANOVA) was performed to study the effect of the studied variables on the dye adsorption (Table 5). A high degree of correlation between experimental and predicted values was obtained according to the R^2^ value (99.97%). The highest adsorbed dye (%) was obtained using HT and the minimum levels of mordant and surfactant and maximum level of silane (Table 2). On the other hand, the lowest LD adsorption (%) was achieved with MMT and the minimum values of the three modifiers used. These results suggest that the nanoclay structure used for LD adsorption (MMT or HT) strongly affects (*p* < 0.05) the lemon hybrid pigment synthesis, as it can be seen in the Pareto’s plot (Figure 1a). Moreover, two significant (*p* < 0.05) positive interactions, mordant-nanoclay and surfactant-nanoclay, were observed.

Depending on the nanoclay structure, the concentration used for the mordant salt and surfactant could favor or inhibit LD adsorption. As it can be seen in Figure 1b, HT synthesis achieved better results when the minimum concentrations of surfactant (SDS) and the mordant salts were used. However, with MMT it was better to increase the concentration of all the used modifiers (CPB and mordant). Consequently, the best synthesis conditions to maximize LD adsorption were defined as 5 wt% of surfactant (SDS), 5 wt% of mordant and 50% (*v/v*) ethanol as solvent for HT; and 10 wt% of surfactant (CPB), 5 wt% of mordant, 1 wt% of silane and 100% distilled water as solvent for MMT.

It is well known that the adsorption process is dependent on the natural dye structure used [37]. LD presents anionic characteristics [8] and the ability of HT to host anionic dyes has been reported by other authors. Bascialla and Regazzoni proposed that sorption of anionic dyes by HT mainly takes place by intercalation of the dye by anion exchange, where the intercalated dye anions stand perpendicular to the nanoclay layers [39]. Moreover, the photostability of anionic natural dyes can be improved by intercalation into the HT layer, if the dye has a hydrophilic nature and a rather planar structure. In addition, contribution of the electrostatic interaction between the positively charged HT layer and the intercalated anionic dye should also be considered [16].

The presence of surfactant, mordant and silane modifiers allowed LD adsorption in both nanoclays, due to nanoclay interlayer and surface modifications [37]. Sheet clay polarity has also been reported to influence the encapsulation of the organic dye [4]. Surface modifiers such as surfactants or silane-coupling agents are needed to modify the nanoclay surface polarity. The π–π interaction between natural dyes and the benzene ring of surfactants has been reported to contribute to enhance the stability of the organoclays [4]. Additionally, the presence of surfactants could modify the interlayer space and electrostatic interactions between HT and natural dyes [19]. Mordant salts could act as anchoring agents (formation of coordinate bonds) improving fixation of natural dyes, the interaction of natural dyes with inorganic nanoclays and color fastness [40].

### 3.2. Lemon Hybrid EOs

#### 3.2.1. Hybrid Systems with 100 wt% LEO

The synthesis performance of lemon hybrid EO systems was studied by using 100 wt% LEO with HT and MMT nanoclays. The limonene adsorption in the separated solvents after functionalization was determined at 220 nm and 265 nm. In this way, the best synthesis conditions were correlated to a less amount of limonene found in these supernatants.

**Figure 2:**
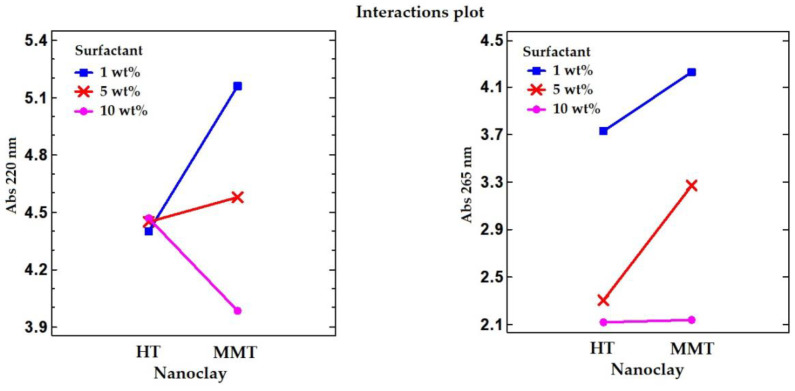
Interactions plot between type of nanoclay (HT and MMT) and surfactant concentration (SDS for HT or CPB, for MMT at 1, 5, 10 wt%) with limonene absorption at 220 nm (**left**) and 265 nm (**right**), in the separated solvents.

Figure 2 shows the interactions plot obtained between type of nanoclays (X axis) and each level of surfactant concentrations: 1, 5, and 10 wt%; the Y axis is the dependent variable, the limonene absorption at the two studied wavelengths (220 and 265 nm). These plots show the relationship between the type of nanoclay and the limonene absorption, which depends on the value of the surfactant concentration. The lines for the surfactant concentrations are not parallel, indicating that there is an interaction between surfactant concentrations and type of nanoclay on limonene absorption.

At 220 nm, no significant effect (*p* > 0.05) was observed for SDS concentration and the amount of limonene found in the supernatant for HT. However, for MMT, the CPB concentration used showed a significant effect (*p* < 0.05) with a decrease in absorbance with increasing surfactant concentration. According to these results, the maximum LEO adsorption was achieved using MMT clay and 10 wt% of CPB, indicating the presence of LEO in the nanoclay structure. At lower energies, 265 nm, a strong significant (*p* < 0.05) interaction between both nanoclays and the surfactant concentration was also found (Figure 2), following a similar trend than that observed for MMT clay at 220 nm. The line for 10 wt% of surfactants is flat at 265 nm, noting that, among these limonene absorptions, there is no difference between the type of nanoclay and 10 wt% of surfactants. In contrast, the lines for 1 and 5 wt% slope up to the right, indicating that, among these limonene absorptions at 265 nm, the HT has lower limonene absorptions than the MMT group.

In conclusion, the best results obtained for both nanoclays were found using the maximum amount of surfactant, SDS or CPB (samples MMT.EO.3 and HT.EO.3, Table 3), allowing good interactions between LEO and the nanoclay. So, it seemed that a high concentration of surfactants was needed to effectively modify the nanoclays interlayer and surface resulting in an increase in LEO adsorption.

Giannakas et al. [24] used a “ModifiedClay (MC)” method to adsorb thymol EO into MMT to obtain hybrid EO systems for the preparation of nanocomposite films with improved tensile strength, water swelling and barrier properties to be used as active food packaging materials. Similar results were found by other authors when different EOs were incorporated into MMT [41,42,43]. In addition, Campos-Requena et al. [44] and Pola et al. [25] also reported the mix of the surfactant and EO before their incorporation into the nanoclays.

#### 3.2.2. Hybrid Systems with 300 wt% LEO

**Figure 3:**
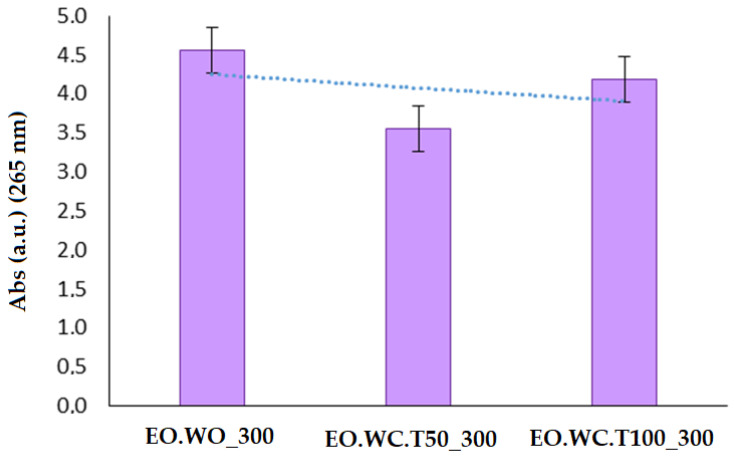
Maximum absorption at 265 nm obtained for separated solvents from EO.WO_300, EO.WC.T50_300 and EO.WCT100_300 samples (Table 4).

MMT and 10 wt% CPB were used to evaluate the effect of three different experimental procedures (Table 4) to adsorb 300 wt% LEO. This LEO concentration was selected in order to ensure a final lemon fragrance in the developed bio-nanocomposites. The separated solvents obtained for the three samples tested at 300 wt% loading were analyzed by UV-Vis spectroscopy to evaluate the synthesis performance, obtaining a lower absorbance for EO.WC.T50_300 sample (Figure 3). According to these results, it seems that the use of an evaporation/adsorption process in a closed recipient at 50 °C during the exchange step had a positive effect by improving the MMT adsorption capacity. No significant differences were found between the synthesis performed at room temperature and the vapor adsorption at 100 °C (Figure 3).

According to some authors [17], the use of an evaporation/adsorption process without the addition of organic solvents could be applied to obtain good results due to the advantage of the EOs evaporation and the use of closed recipients to achieve the vapor adsorption of EOs into MMT clays, previously modified with surfactants. The use of 50 °C and water dispersions or just the EO at 120 °C in closed recipients have been reported [17].

### 3.3. Bionanocomposites Characterization

#### 3.3.1. Bionanocomposites with Lemon Hybrid Pigments

The optimized lemon hybrid pigments with HT and MMT were incorporated into INZEA biopolymer for the development and characterization of bio-nanocomposites at different hybrid pigments loading (3, 5, and 7 wt%). Blank samples from MMT and HT pure nanoclays were also extruded and used to compare color, thermal, and mechanical properties of the developed bio-nanocomposites.

**Figure 4:**
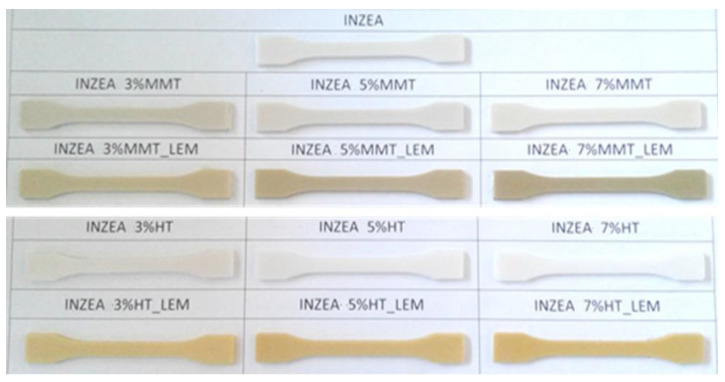
Polyester-based matrix (INZEA)-based bio-nanocomposites obtained at different lemon hybrid pigments loading (3, 5, and 7 wt%).

The selection of the proposed weight amounts was done considering that, regardless of the final color of the samples, enhancement in mechanical performance in polymer-based composites is generally obtained at similar contents, where the agglomeration of the nanoscale additives is usually limited. Visual images of the produced materials are reported in Figure 4. A darker color was evidenced for MMT containing samples due to the intrinsic color of this nanoclay compared to HT. The results obtained for color parameters are included in Table 6. A positive correlation between the nanoclay type, its content and the dye absorbed by the nanocomposites was evidenced. As already observed by other authors, the disperse dyeing behavior of composites depends on the dye’s affinity to the polymer, chemical structure of the dispersed dye, and active area [45].

Previous research articles have focused on the dyeing properties of polypropylene and polyamide six-layered clay incorporated in nanocomposites prepared by melt compounding. Toshniwal et al. [46] suggested that polypropylene fibers could be made dyeable with disperse dyes by the addition of nanoclay particles in the polymer matrix. Another research work done by Razafimahefa et al. [47] showed that the introduction of nanoclays improved the dyeing ability of nylon with dispersed dyes.

In the case of INZEA-based samples, the neat matrix was characterized by a high lightness value (Table 6), induced by the color of the neat polymer. The addition of MMT and HT in INZEA-based systems produced, respectively, a reduction and increase in L* values. This phenomenon was due to the MMT and HT powders’ intrinsic colors, which were able to modify the final aesthetic quality and appearance of the different samples. The L* value was maintained stable with increasing the ratio nanofiller/polymer. Moreover, no variation of a* and b* values was observed with increasing the amount of MMT and HT in the INZEA_MMT and INZEA_HT-based systems (Table 6).

The presence of lemon hybrid pigments in INZEA-based systems determined a remarkable variation in CIELAB values. In INZEA MMT_LEM-based systems, a reduction in L* and increase in b* values, due to the color of the bio-based nanofillers, were observed. Lemon pigments, in terms of CIELAB parameters, were influenced by the color of the lemon skin from which they were extracted [48]. A reduction in lightness and an increase in a* and b* coordinates were registered with loading INZEA with HT_LEM nanofiller. The influence of MMT_LEM and HT_LEM hybrid pigments were more visible with increasing the nanofiller ratio. Comparing INZEA MMT_LEM and INZEA HT_LEM, it is possible to highlight that HT_LEM-based samples were characterized by a bright yellow color compared to MMT_LEM samples. Essentially, the values registered for b* parameter and INZEA HT_LEM were higher than those obtained for INZEA MMT_LEM samples. Lastly, gloss values were reduced by using unmodified MMT and HT fillers or using MMT_LEM and HT_LEM pigments, this variation were more evident increasing the nanofiller content. A similar behavior has been reported for different polymeric matrices and nanofillers [49].

Tensile characterization results of INZEA-based samples are reported in Table 7. In general, a decrease in terms of strength and strain at break was observed for all formulations, regardless of the type or weight amount of the nanopigment introduced in the polymer matrix. Specifically, tensile strength maintained an acceptable deformation level only when 3 wt% of unmodified MMT was considered, while HT, even at the same lower amount, strongly decreased the ductility of the matrix. It has been reported [50] that a dye−clay nanopigment, synthesized via a cationic exchange reaction between mineral nanoclays and organic dyes, can be considered to gain the advantages of nanoclay reinforcement and colorant features, simultaneously. Nevertheless, even if the dispersion in polymer matrices can offer superior colorimetric properties and color fastness compared to the inorganic counterparts, mechanical enhancement can be obtained only if the nanopigment has been properly intercalated or exfoliated in the formulation. In our case, the probable formation of some aggregates and a limited extent of intercalation/exfoliation of MMT and HT in the polymer matrix could explain the observed results. This observation is strictly correlated with the experimental results, which showed a strong decrease in ductility, in parallel with a limited reinforcement effect (values of elastic modulus were essentially maintained constant for all formulations). As already observed by Marchante et al. in the case of EVA-based systems [51], a decrease in elastic modulus (E) could be related to an overall decrease in the rigidity provided by the nanoclay, as the specific surface area was decreased and the stress was transferred through the matrix to the nanoclay. On the other hand, almost all of the samples presented lower deformation values at break (ε_b_). In this case, the presence of tactoids could reduce the capacity of the material to absorb energy as a consequence of a reduction in the polymer’s mobility chain. The strain at break also decreased when the amount of the nanoadditive increased and this reduction was more noticeable in samples with nanopigments compared to samples with unmodified nanoclays, confirming that the added nanopigments reduced the compatibility between MMT (and HT) and the polyester-based matrix [52].

**Figure 5:**
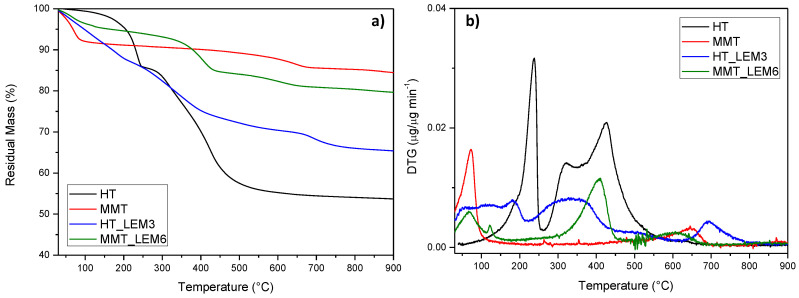
(**a**) TG (**a**) and DTG (**b**) results obtained for HT, MMT and lemon hybrid pigments (HT-LEM and MMT-LEM).

Thermal characterization of INZEA-based formulations containing the lemon hybrid pigments was also performed, by considering the overall stability of the pigmented nanoclays during the heating occurring in the extrusion phase. TGA analysis in nitrogen atmosphere of MMT_LEM and HT_LEM samples was performed and the resulting TG (a) and DTG (b) curves are presented in Figure 5. In particular, TGA for neat nanoclays and hybrid materials were reported. According to the literature, the first step observed in the TGA curves, up to 200° C, was attributed to the desorption of water molecules from MMT’s pores (non constitutional water) and between the layers. A further weight loss above 400 °C was attributed to the dihydroxylation of the remaining OH groups of MMT. The final residue corresponded to the thermal degradation of the phyllosilicate into an amorphous phase [53]. In the case of HT, the weight loss curve can be divided into three main thermal steps. The first one was associated with the loss of hydration water occurring below 200 °C, followed by the loss of hydroxyls between 200 and 600 °C [54]. Since natural dyes are susceptible to temperature, the evaluation of their thermal stability is an important issue to be considered for their correct functional use in thermally processed materials.

Hybrid systems showed a different thermal behavior according to the chemical nature of the nanoclay used [55,56]. In the case of MMT_LEM, the hybrid compound exhibited a thermal behavior consisting of the dehydration of the compound (loss of water adsorbed and intercalated into the MMT), followed by a weak shoulder event centered at 122 °C). After that, the decomposition of the organic dye occurred, starting at about 260 °C and completing at nearly 460 °C. Nevertheless, the overall stability of the modified MMT was maintained, at least in the temperature frame of the polyester-based matrix selected for the present study [57]. On the other hand, the presence of the lemon dye between HT layers significantly affected the thermal behavior of the nanoclay in the lower temperature range, since a distinct weight loss was observed (with a residual weight at 200 °C of 87% and 95%, respectively, for HT_LEM and unmodified HT). So, even if HT was thermally stabilized in the presence of the dye (residual mass at 900 °C was 65% and 54%, respectively, for HT_LEM and unmodified HT), it was easily degradable at the processing temperature range of the selected matrix [16].

**Figure 6:**
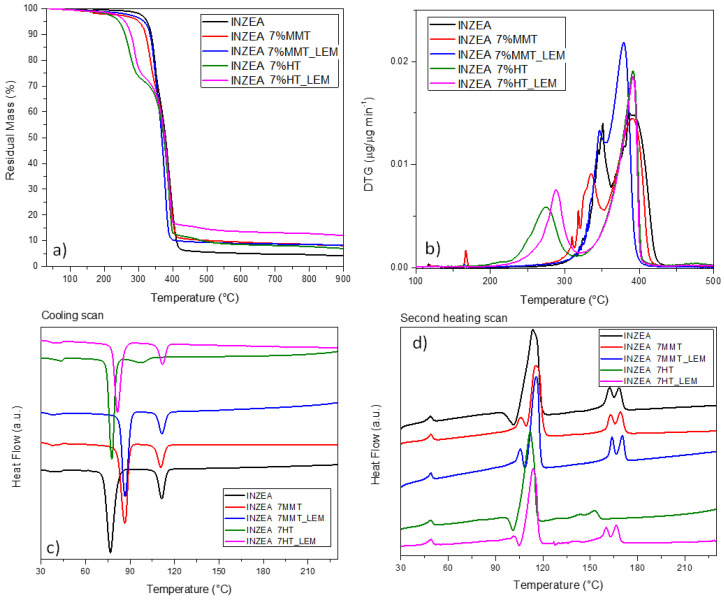
TG (**a**) and DTG (**b**) profiles and DSC curves, cooling (**c**) and 2nd heating (**d**) for INZEA containing MMT and HT functionalized with lemon hybrid pigments.

The observed behavior for lemon hybrid pigments greatly influenced also the response of INZEA-based systems. In particular, the curves related to mass loss (a) and derivative weight loss (b) for the system containing 7 wt% of nanopigments (representative of the same trend observed for the formulations containing 3 and 5 wt% of nanopigments) are reported in Figure 6a,b. As it has been reported [58], a double degradation peak can be found for the INZEA neat matrix, with two main steps centered at 350 and 400 °C, that could match with the possible degradation temperatures of PLA and poly(butylene succinate) (PBS) polyesters, respectively. It is also important to note that at 900 °C, unmodified INZEA matrix maintained a residual mass of ca. 5 wt%, which was increased when the nanofillers were introduced. This behavior is in accordance with the possible presence of an inorganic filler initially in the formulation of the commercial product.

A deep analysis of the DTG curves for the systems containing pure HT or MMT and the lemon hybrid pigments clearly indicated that, in the case of HT, the thermal stability of the overall blend was strongly affected. Specifically, while the T_peak2_ remained substantially unaffected (only a small variation in the degradation rate was noted), the T_peak1_ for the less stable component was shifted from 350 °C for neat INZEA to 287 and 275 °C, respectively, for INZEA 7%HT_and INZEA 7%HT_LEM. An analogous behavior, even if limited in the shift values, was noted for MMT containing materials, where T_peak1_ for the less stable component shifted to 346 and 335 °C, respectively, for INZEA 7%MMT and INZEA 7%MMT_LEM. In the case of MMT loading, a shift towards lower temperatures was even measured for the more stable component of the blend (from 392 to 379 °C for INZEA 7%MMT_LEM).

In accordance with the literature, this degradation path, differing from that of the neat matrix, can be rationalized by considering the organic nature of the nanopigments, that were indeed responsible of the decrease in thermal stability of the polyester matrix [36]. The reduction in the thermal stability in the first step of the decomposition process of INZEA can be justified by the fact that unmodified nanoclay could catalyze the hydrolysis reaction, MMT platelets having –OH moieties on the surface promoting degradation [59] and organo-modified Al layered double hydroxide (LDH) for the presence of moisture in the interlayer spacing [60].

As previously reported by Wei et al. [61], the presence of residual metals, such as Al, Fe, Zn and Sn, in LDH could cause an apparent thermal degradation of the polyester component, catalyzing the inter- and intra-molecular transesterification reactions of the polyesters. On the other hand, the decrease in thermal stability can be also explained due to the barrier properties of the nanoclay, which depend on the level of exfoliation. Only when the clay layers are exfoliated, they may have reduced the volatilization of the degradation products, and then, the thermal stability could increase [51,62]. In our case, the observed limited mechanical enhancement, due to reduced exfoliation of the nanoclays, can also justify the reduced thermal stability of the same samples.

Figure 6c,d shows the DSC thermograms obtained during the DSC cooling and second heating scans for all formulations. Two different peaks were observed for neat INZEA, indicating the presence of two main polyesters in the polymer matrix, in agreement with the behavior previously observed by Ramos et al [58]. The analysis of the experimental curves indicated that no substantial variations were introduced in the glass transition temperatures, while the main effect was related to the shift of melting and crystallization temperatures, in parallel with the evidence or disappearance of multiple melting peaks. In detail, MMT and HT differently affected the processes: while INZEA MMT and INZEA MMT_LEM hybrid materials both modified the crystallization event, by shifting the T_c_ of the low melting polyester to higher temperatures, the effect on the high melting polyester was not visible. It can be considered that MMT could be located and better dispersed in the PBS phase than in the polylactic fraction, due to the higher interaction between PBS and MMT than that between PLA and MMT [63], being the modification of the melt crystallization event evident only in the PBS fraction. This effect was also reflected in the heat flow curve during the second heating (Figure 6d), where the cold crystallization was progressively inhibited and a double melting peak appeared, confirming the formation of crystals with different thickness, connected with a minute fraction of thinner and/or less perfect crystals originated during cooling and melting of the originally crystallized material [64]. No valuable variations, in terms of temperature and curve shape, were noted in the case of INZEA_7%MMT_LEM.

On the other hand, it was found that the presence of nanopigments intercalated in HT was beneficial to the cooling and melting behavior of the PLA fraction. In detail, while INZEA_7%HT_LEM substantially maintained the same crystallization/melting attitude of the neat polyester blend, the unmodified HT was responsible for a general decrease in melting temperature in the polylactic fraction. It is well known that when the melting endotherm of neat PLA is bimodal, it can be ascribed to the formation of crystals with different degrees of perfection: the first endothermic is related to the melting of cold crystallized PLA chains, whereas the second peak can be attributed to those PLA crystals which have reorganized to more perfect crystals once they found sufficient thermal energy in the course of cold crystallization. In our case, this evidence can be correlated to the limited activity, as nucleating agent, of the HT reinforcement in the polyester blend and to the reduction in PLA molecular weight, due to the presence of surface hydroxyl groups on LDH and/or metal-catalyzed degradation [65,66].

#### 3.3.2. Bionanocomposites with Lemon Hybrid EOs

Analogously to the thermomechanical characterization of INZEA formulations containing lemon hybrid pigments, the evaluation of the main parameters of lemon-hybrid EO containing samples was also performed. Specifically, the role of LEO, at the best synthesis conditions previously identified (100 wt%, 10 wt% surfactant concentration) when it was introduced in the polymer matrix was further investigated. Results of tensile characterization of the produced INZEA-based samples are reported in Table 8. A significant increase in the Young’s modulus of the nanocomposite materials was observed compared to the biopolymer matrix. In particular, the incorporation of 7 wt% of MMT added with LEO (INZEA_7MMT.EO3) showed an average value of 2249 MPa significantly higher (+64.8%) than the neat matrix, that showed a value of E = 1365 MPa. A similar result was observed for the formulation with 7 wt% of HT and LEO (INZEA_7HT.EO.3) which increased the modulus to 2193 MPa (+60.7%). The increase of stiffness in the nanocomposites due to the addition of nanoclays has been widely demonstrated [67]. This increase in the elastic modulus corresponded to a considerable decrease in deformability for both formulations, reducing the deformation at break of the nanocomposites with MMT and HT to 10 and 8%, respectively. This result is indicative of a poor interface bonding between the nanofillers and the matrix, probably due to the scarce compatibility between the biopolymer and the nanoparticles added with LEO. It was demonstrated that the compatibility between a hydrophobic matrix and nanofillers such as MMT and HT is often poor and can be improved through the use of compatibilizers that mitigate the hydrophilic nature of the nanoclays [68]. It was hypothesized that the presence of essential oils and nanofillers could have two antagonistic effects: on the one hand, EO linked to the filler could mitigate the hydrophilicity of the nanoclays by improving matrix/filler compatibility; on the other hand, the presence of free essential oils in the formulation could reduce the mechanical strength of the composites by acting as a plasticizer and intermolecular lubricant [69]. The results show that both the composites with essential oils increase their moduli with respect to the corresponding nanocomposites without EO, suggesting that an improvement of compatibility matrix/filler occurs. In addition, a slight increase in elongation without strength reduction suggests that free essential oils are almost completely absent. The overlap of these effects does not produce substantial changes in the mechanical tensile characteristics of composites with and without EO, except for an increase in the elastic modulus of composites with added EO. It can be seen that the presence of 7 wt% of nanofiller limits the deformability of the composites. The reduction in maximum elongation with respect to the matrix, for both nanocomposites, was responsible of a reduction in both the yield stress and strain at break.

**Figure 7:**
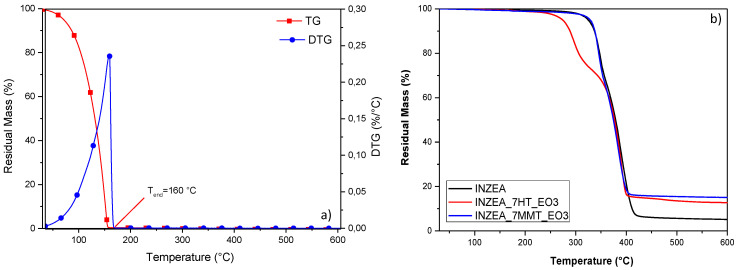
TGA results for pure LEO (**a**) and HT and MMT samples functionalized with 100 wt% LEO (**b**).

The limited, but superior behavior of INZEA_7MMT.EO.3 in terms of deformability, compared to the HT-loaded system, can be justified by considering that the EO containing nanofillers underwent a thermal degradation event when heated during extrusion. In order to justify the narrow effect of the EO in terms of improved ductility, the release of the added EO during a dynamic heating scan, in a TGA inert environment, for INZEA_7MMT.EO.3 and INZEA_7HT.EO.3 was performed. The results of dynamic heating scans are included in Figure 7a,b. In particular, the results of Figure 7a confirmed that the heat stability of the EO was quite limited (the evaporation was completed at 160 °C), but its inclusion into the nanoclays efficiently retained the observed loss during heating of the polyester matrix, especially in the case of MMT (Figure 7b).

In the case of MMT samples functionalized with 300 wt% LEO, the presence of EO.WC.T50 and EO.WC.T100 in INZEA matrix was determined and evaluated, in terms of mechanical performance, by means of tensile tests. Table 9 summarizes the results of mechanical properties obtained for INZEA-based materials in the presence of different fillers (EO.WC.T50 and EO.WC.T100). A clear difference in terms of mechanical properties (decrease in stress and strain (%) at break and improvement in Young’s modulus) was observed. Specifically, the addition of the filler changed completely the mechanical response of the different samples. Neat INZEA showed a ductile behavior which was turned into a fragile behavior in the case of the presence of the nanofillers. A similar behavior was detected in PLA/MMT-based formulations by Othman and co-authors who observed that a high concentration of MMT in PLA changed the ductility of the polyester [70]. No particular variations were observed between the different INZEA+7%EO.WC.T50 and INZEA+7%EO.WC.T100-based materials. This phenomenon highlights that the presence of LEO at the different concentrations used resulted ineffective in terms of improving ductility, but only an increase in stiffness was revealed.

**Figure 8:**
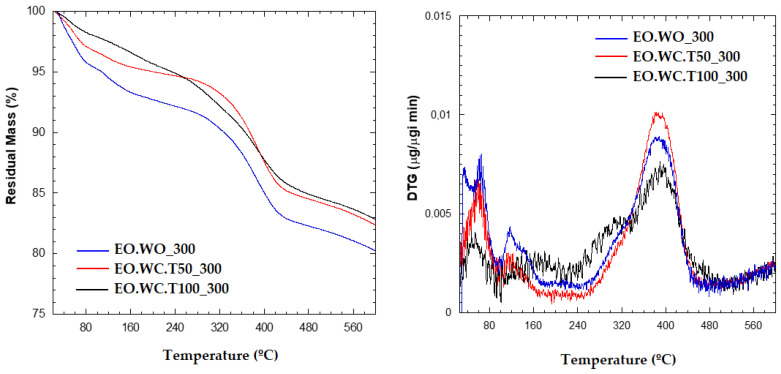
TGA results obtained for lemon hybrid EO systems with MMT at 300 wt% of LEO.

**Figure 9:**
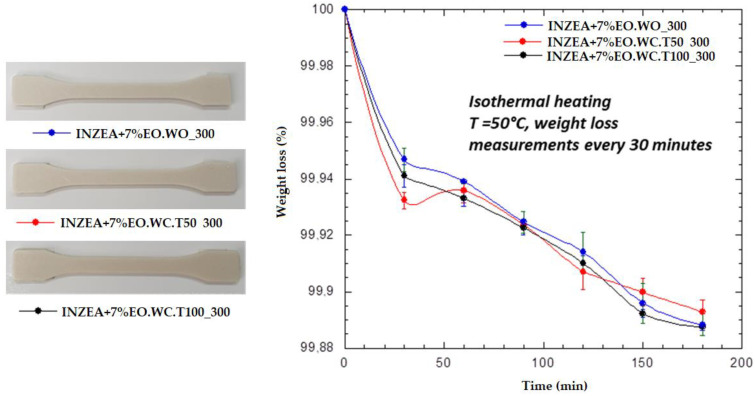
Visual images of samples and TGA results obtained for INZEA-based bio-nanocomposites including MMT hybrid samples functionalized with 300 wt% LEO.

With the aim of evaluating the presence of LEO in the polymer matrix, INZEA+7%EO_W0_300, INZEA+7%EO_WC.T50_300, and INZEA+7%EO_WC.T100_300 samples were tested. First, the thermal stability of the obtained samples was studied by TGA (Figure 8); sample EO.WC_T100_300 exhibited the lower weight loss up to 200 °C, being the more thermally stable in the entire temperature range. The release of LEO during thermal processing was also verified by heating the bio-nanocomposites by TGA isothermally at 50 °C. Weight measurements were taken every 30 min and the obtained results (Figure 9) indicated quite similar kinetics for LEO release in all samples, although these tests should be run for a longer time. Therefore, the obtained results for the developed LEO hybrid systems with MMT confirmed that LEO release during heating was better controlled in the case of sample EO_WC_T100_300, which was also the one showing a higher final lemon smell compared to the other ones.

## 4. Conclusions

Lemon waste hybrid pigments and EOs were successfully synthetized using MMT and CHT nanoclays as hosts for dye and EO adsorption. The best synthesis conditions to ensure the maximum adsorption of lemon dye were found using 50% (*v/v*) ethanol and distilled water with HT and MMT, respectively. The modifier contents used were optimized by using statistical design of experiments and they were influenced by the nanoclay type: 5 wt% of surfactant (SDS) and 5 wt% of aluminum salt as mordant were used for HT; while 10 wt% CPB, 5 wt% of aluminum salt as mordant and 1 wt% of silane were used for MMT. Interesting colors were achieved with the addition of nanopigments to a polyester-based matrix (INZEA), in particular at 7 wt% loading. However, some decrease in mechanical and thermal properties was observed for the obtained nanocomposites which were linked to some nanopigment agglomeration and limited nanoclay exfoliation in the polymer matrix. A significant effect of the surfactant concentration used to synthetize the lemon hybrid EOs was observed. Surfactant (10 wt%) was fixed and the best synthesis performance was found with MMT nanoclay. The EO content was increased to 300 wt% and an evaporation/adsorption process at 50 °C was performed. The INZEA-based nanocomposites added with lemon hybrid EO showed interesting odorous properties, increasing the thermal stability of the lemon EO by its inclusion into the nanoclay.

In conclusion, lemon waste dye and EO incorporated into nanoclays have demonstrated their potential to be added in polyester-based nanocomposites as coloring and odor additives with a limited reinforcing effect. Further work will be needed to improve the interaction between the polymer matrix and the nanoclays. In addition, organoleptic behavior, release kinetics and antioxidant/antimicrobial properties are other important issues that would be also necessary to be evaluated to improve the functionality of the obtained formulations.

## Figures and Tables

**Table 1:** Independent variables and selected levels used in the 2^4−1^ fractional experimental design.

Independent Variables	−1	+1
Nanoclay structure	MMT	HT
Surfactant concentration (wt%)	5	10
Mordant concentration (wt%)	5	10
Silane concentration (wt%)	0	1

**Table 2:** 2^4−1^ fractional experimental design matrix, additional experiments performed with water as solvent and lemon dye adsorbed over the initially added dye (%).

Experiment	Nanoclay	Surfactant (wt%) *	Mordant (wt%)	Silane (wt%)	Adsorbed dye (%)	Solvent
1	HT	10	10	1	99.78	50% (*v/v*) ethanol
2	MMT	10	10	0	98.99	
3	HT	5	10	0	99.84	
4	MMT	5	10	1	98.97	
5	HT	10	5	0	99.88	
6	MMT	10	5	1	98.94	
7	HT	5	5	1	99.94	
8	MMT	5	5	0	98.87	
2w	MMT	10	10	0	99.45	Distilled water
4w	MMT	5	10	1	99.44	
6w	MMT	10	5	1	99.59	
8w	MMT	5	5	0	99.38	

* Cetylpyridinium bromide (CPB) was used for montmorillonite (MMT) and sodium dodecyl sulfate (SDS) for hydrotalcite (HT).

**Table 3:** 2^1^•3^1^ fractional experimental design matrix and responses obtained for lemon essential oil (LEO) adsorption at 100 wt%.

Sample Code	Independent Variables		Response		Solvent
	Nanoclay	Surfactant (wt%) *	Absorbance 220 nm	Absorbance 265 nm	
HT.EO.1	HT	1	4.39	3.73	50% (*v/v*) ethanol
HT.EO.2	HT	5	4.45	2.30	
HT.EO.3	HT	10	4.47	2.11	
MMT.EO.1	MMT	1	5.16	4.23	Distilled water
MMT.EO.2	MMT	5	4.58	3.27	
MMT.EO.3	MMT	10	3.98	2.13	

* CPB was used for MMT and SDS for HT.

**Table 4:** Experimental tests performed with 300 wt% LEO, MMT and 10 wt% of CPB.

Sample Code	Dispersion Conditions	Temperature (°C)	LEO (wt%)
EO.WO_300	Open vessel, water	Ambient/Room	300
EO.WC.T50_300	Closed vessel, water	50	300
EO.WC.T100_300	Closed, water *	100	300

* Water was used in the first step with CPB and MMT. No solvent was used for LEO addition to the clay paste.

**Table 5:** Variance analysis for lemon dye synthesis performance.

Factor	Sum of Squares	DF	Mean Square	F-Value	*p*-Value
A (Nanoclay)	0.1875	1	0.1875	860.35	**0.0012**
C (Mordant)	0.0004	1	0.0004	1.77	0.3145
D (Silane)	0.0002	1	0.0002	0.72	0.4858
AB (Nanoclay-Surfactant)	0.0054	1	0.0054	24.77	**0.0381**
AC (Nanoclay-Mordant)	0.0154	1	0.0154	70.66	**0.0139**
Total Error	0.0004	2	0.0002		
Total (corr.)	1.6982	7			
R^2^ (%) = 99.97					
Adj R^2^ (%) = 99.91					

**Table 6:** CIELAB parameters for INZEA bio-nanocomposites with MMT and HT lemon hybrid pigments (m ± SD; *n* = 3).

Formulation	L*	a*	b*	∆E*	Gloss (°)
White Control	99.47 ± 0.00	−0.08 ± 0.01	−0.08 ± 0.01	-	121 ± 0
INZEA	81.66 ± 0.32	0.65 ± 0.05	5.11 ± 0.04	18.56 ± 0.3	68 ± 3
INZEA 3%MMT	75.98 ± 0.50	1.17 ± 0.06	8.01 ± 0.29	24.87 ± 0.56	69 ± 3
INZEA 3%MMT_LEM	73.72 ± 0.73	0.40 ± 0.07	18.07 ± 0.30	31.51 ± 0.74	54 ± 4
INZEA 3%HT	84.53 ± 0.89	0.26 ± 0.08	4.46 ± 0.07	15.62 ± 0.87	66 ± 3
INZEA 3% HT_LEM	77.38 ± 0.51	3.32 ± 0.31	31.10 ± 0.86	38.37 ± 1.01	55 ± 3
INZEA 5%MMT	78.70 ± 0.76	0.84 ± 0.08	8.08 ± 0.22	22.34 ± 0.78	65 ± 4
INZEA 5%MMT_LEM	67.42 ± 0.29	0.54 ± 0.06	20.13 ± 0.07	37.89 ± 0.21	64 ± 1
INZEA 5% HT	86.09 ± 0.12	0.16 ± 0.04	4.36 ± 0.07	14.11 ± 0.13	62 ± 2
INZEA 5% HT _LEM	73.52 ± 0.33	5.22 ± 0.14	33.53 ± 0.51	42.80 ± 0.35	55 ± 1
INZEA 7%MMT	79.50 ± 0.74	0.90 ± 0.07	8.83 ± 0.16	21.89 ± 0.66	60 ± 5
INZEA 7%MMT_LEM	62.37 ± 0.38	0.85 ± 0.04	20.80 ± 0.04	42.58 ± 0.03	53 ± 1
INZEA 7% HT	86.22 ± 0.19	0.14 ± 0.04	4.22 ± 0.08	13.93 ± 0.19	57 ± 1
INZEA 7% HT _LEM	71.18 ± 0.33	6.09 ± 0.35	35.87 ± 0.57	46.16 ± 0.69	50 ± 1

**Table 7:** Mechanical parameters for INZEA bio-nanocomposites with MMR lemon hybrid pigment (MMT_LEM) and HT lemon hybrid pigment (HT_LEM) nanofillers at 3 different concentrations (m ± SD; *n* = 5).

Formulation	σ_b_ (MPa)	ε_b_ (%)	E (MPa)
INZEA	38 ± 1	250.5 ± 5.0	1365 ± 71
INZEA 3%MMT	35 ± 2	194.1 ± 17.2	1328 ± 38
INZEA 3%MMT_LEM	27 ± 10	18.1 ± 15.7	1504 ± 226
INZEA 3%HT	26 ± 1	56.3 ± 11.9	1275 ± 71
INZEA 3%HT_LEM3	35 ± 2	10.6 ± 1.3	1275 ± 49
INZEA 5%MMT	34 ± 2	10.5 ± 1.1	1281 ± 123
INZEA 5%MMT_LEM	33 ± 3	11.1 ± 1.9	1212 ± 37
INZEA 5%HT	34 ± 2	8.8 ± 1.4	1195 ± 48
INZEA 5%HT_LEM	30 ± 2	5.6 ± 0.9	1311 ± 52
INZEA 7%MMT	34 ± 1	7.2 ± 0.3	1331 ± 28
INZEA 7%MMT_LEM	32 ± 4	7.5 ± 1.4	1397 ± 226
INZEA 7%HT	33 ± 2	8.2 ± 0.6	1219 ± 29
INZEA 7%HT_LEM	33 ± 3	4.4 ± 0.3	1649 ± 396

**Table 8:** Mechanical parameters obtained for INZEA bio-nanocomposites with MMT and HT lemon-hybrid essential oil (EO) (m ± SD; *n* = 5).

Formulation		σ_b_ (MPa)	ε_b_ (%)	E (MPa)
INZEA	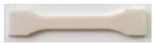	38 ± 1	250.5 ± 5.0	1365 ± 71
INZEA 7%MMT		34 ± 1	7.2 ± 0.3	1331 ± 28
INZEA_7MMT.EO.3	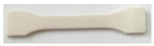	33 ± 3	10.0 ± 1.0	2249 ± 19
INZEA 7%HT		33 ± 2	8.2 ± 0.6	1219 ± 29
INZEA_7HT.EO.3	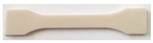	35 ± 3	8.0 ± 1.0	2193 ± 28

σ_b_ (MPa): Strength at break; ε_b_ (%) at σ_b_: Strain at break; E (MPa): Young’s Modulus.

**Table 9:** Mechanical parameters for INZEA bio-nanocomposites with MMT samples functionalized with 300 wt% LEO (m ± SD; *n* = 5).

Formulation	σ_b_ (MPa)	ε_b_ (%)	E (MPa)
INZEA	38 ± 1	250.5 ± 5.0	1365 ± 71
INZEA+7%EO.WC.T50_300	36 ± 3	7.0 ± 1.0	1898 ± 42
INZEA+7%EO.WC.T100_300	35 ± 2	7.0 ± 1.0	1889 ± 44
INZEA+7%EO.W0_300	35 ± 2	6.0 ± 1.0	1764 ± 34

σ_b_ (MPa): Strength at break; ε_b_ (%) at σ_b_: Strain at break; E (MPa): Young’s Modulus.

## References

[1] Rozin P., Spranca M., Krieger Z., Neuhaus R., Surillo D., Swerdlin A., Wood K. (2004). Preference for natural: Instrumental and ideational/moral motivations, and the contrast between foods and medicines. Appetite.

[2] Amchova P., Kotolova H., Ruda-Kucerova J. (2015). Health safety issues of synthetic food colorants. Regul. Toxicol. Pharmacol..

[3] Zerin I., Farzana N., Sayem A.S.M., Anang D.M., Haider J. (2020). Potentials of Natural Dyes for Textile Applications. Encycl. Renew. Sustain. Mater..

[4] Micó-Vicent B., Jordán J., Perales E., Martínez-Verdú F., Cases F. (2019). Finding the Additives Incorporation Moment in Hybrid Natural Pigments Synthesis to Improve Bioresin Properties. Coatings.

[5] Ebrahimi I., Parvinzadeh Gashti M. (2016). Extraction of polyphenolic dyes from henna, pomegranate rind, and *Pterocarya fraxinifolia* for nylon 6 dyeing. Color. Technol..

[6] Jaffer M., Shaheen S., Ashraf H., Hanif U. (2019). Green vegetation: A promising source of color dyes. Green Process. Synth..

[7] Del Río J.A., Fuster M.D., Gómez P., Porras I., García-Lidón A., Ortuño A. (2004). Citrus limon: A source of flavonoids of pharmaceutical interest. Food Chem..

[8] Zhang Y., Wan H., Zhao J., Li J. (2019). Biosorption of anionic and cationic dyes via raw and chitosan oligosaccharide-modified Huai Flos Chrysanthemum at different temperatures. RSC Adv..

[9] Delgado-Vargas F., Jiménez A.R., Paredes-López O. (2000). Natural pigments: Carotenoids, anthocyanins, and betalains—characteristics, biosynthesis, processing, and stability. Crit. Rev. Food Sci. Nutr..

[10] Downham A., Collins P. (2000). Colouring our foods in the last and next millennium. Int. J. food Sci. Technol..

[11] Burt S. (2004). Essential oils: Their antibacterial properties and potential applications in foods—a review. Int. J. Food Microbiol..

[12] Nakhli A., Mbouga M.G.N., Bergaoui M., Khalfaoui M., Cretin M., Huguet P. (2018). Modeling of essential oils adsorption onto clays towards a better understanding of their interactions. J. Mol. Liq..

[13] Chakraborty C., Dana K., Malik S. (2011). Intercalation of perylenediimide dye into LDH clays: Enhancement of photostability. J. Phys. Chem. C.

[14] Chakraborty C., Dana K., Malik S. (2012). Lamination of cationic perylene in montmorillonite nano-gallery: Induced J-aggregated nanostructure with enhanced photophysical and thermogravimetric aspect. J. Phys. Chem. C.

[15] Kohno Y., Kinoshita R., Ikoma S., Yoda K., Shibata M., Matsushima R., Tomita Y., Maeda Y., Kobayashi K. (2009). Stabilization of natural anthocyanin by intercalation into montmorillonite. Appl. Clay Sci..

[16] Kohno Y., Totsuka K., Ikoma S., Yoda K., Shibata M., Matsushima R., Tomita Y., Maeda Y., Kobayashi K. (2009). Photostability enhancement of anionic natural dye by intercalation into hydrotalcite. J. Colloid Interface Sci..

[17] Giannakas A., Tsagkalias I., Achilias D.S., Ladavos A. (2017). A novel method for the preparation of inorganic and organo-modified montmorillonite essential oil hybrids. Appl. Clay Sci..

[18] Kohno Y., Inagawa M., Ikoma S., Shibata M., Matsushima R., Fukuhara C., Tomita Y., Maeda Y., Kobayashi K. (2011). Stabilization of a hydrophobic natural dye by intercalation into organo-montmorillonite. Appl. Clay Sci..

[19] Kohno Y., Asai S., Shibata M., Fukuhara C., Maeda Y., Tomita Y., Kobayashi K. (2014). Improved photostability of hydrophobic natural dye incorporated in organo-modified hydrotalcite. J. Phys. Chem. Solids.

[20] Guillermin D., Debroise T., Trigueiro P., de Viguerie L., Rigaud B., Morlet-Savary F., Balme S., Janot J.-M., Tielens F., Michot L. (2019). New pigments based on carminic acid and smectites: A molecular investigation. Dye. Pigment..

[21] El-Mougy N.S. (2009). Effect of some essential oils for limiting early blight (Alternaria solani) development in potato field. J. Plant Prot. Res..

[22] Campos-Requena V.H., Rivas B.L., Pérez M.A., Figueroa C.R., Figueroa N.E., Sanfuentes E.A. (2017). Thermoplastic starch/clay nanocomposites loaded with essential oil constituents as packaging for strawberries − In vivo antimicrobial synergy over Botrytis cinerea. Postharvest Biol. Technol..

[23] Su H.-J., Chao C.-J., Chang H.-Y., Wu P.-C. (2007). The effects of evaporating essential oils on indoor air quality. Atmos. Environ..

[24] Giannakas A., Stathopoulou P., Tsiamis G., Salmas C. (2020). The effect of different preparation methods on the development of chitosan/thyme oil/montmorillonite nanocomposite active packaging films. J. Food Process. Preserv..

[25] Pola C.C., Medeiros E.A.A.A., Pereira O.L., Souza V.G.L.L., Otoni C.G., Camilloto G.P., Soares N.F.F.F. (2016). Cellulose acetate active films incorporated with oregano (Origanum vulgare) essential oil and organophilic montmorillonite clay control the growth of phytopathogenic fungi. Food Packag. Shelf Life.

[26] Bernardos A., Bozik M., Alvarez S., Saskova M., Perez-Esteve E., Kloucek P., Lhotka M., Frankova A., Martinez-Manez R. (2019). The efficacy of essential oil components loaded into montmorillonite against Aspergillus niger and Staphylococcus aureus. Flavour Fragr. J..

[27] Hammoudi N., Ziani Cherif H., Borsali F., Benmansour K., Meghezzi A. (2020). Preparation of active antimicrobial and antifungal alginate-montmorillonite/lemon essential oil nanocomposite films. Mater. Technol..

[28] Park H.-M., Lee W.-K., Park C.-Y., Cho W.-J., Ha C.-S. (2003). Environmentally friendly polymer hybrids Part I Mechanical, thermal, and barrier properties of thermoplastic starch/clay nanocomposites. J. Mater. Sci..

[29] Usuki A., Hasegawa N., Kato M., Kobayashi S. (2005). Polymer-clay nanocomposites. Inorganic Polymeric Nanocomposites and Membranes.

[30] Bee S.-L., Abdullah M.A.A., Bee S.-T., Sin L.T., Rahmat A.R. (2018). Polymer nanocomposites based on silylated-montmorillonite: A review. Prog. Polym. Sci..

[31] Lakshmi M.S., Narmadha B., Reddy B.S.R. (2008). Enhanced thermal stability and structural characteristics of different MMT-Clay/epoxy-nanocomposite materials. Polym. Degrad. Stab..

[32] Tornuk F., Sagdic O., Hancer M., Yetim H. (2018). Development of LLDPE based active nanocomposite films with nanoclays impregnated with volatile compounds. Food Res. Int..

[33] Pollet E., Avérous L. (2011). Production, chemistry and properties of polyhydroxyalkanoates. Biopolym. Mater. Sustain. Film. Coat..

[34] Y S., Rao P. (2019). Material conservation and surface coating enhancement with starch-pectin biopolymer blend: A way towards green. Surfaces and Interfaces.

[35] Roy I., Visakh P.M. (2014). Polyhydroxyalkanoate (PHA) Based Blends, Composites and Nanocomposites.

[36] Marchante V., Martínez-Verdú F., Beltrán M., Gomis A. (2012). Mechanical, thermal and colorimetric properties of LLDPE coloured with a blue nanopigment and conventional blue pigments. Pigment Resin Technol..

[37] Micó-Vicent B., Jordán J., Martínez-Verdú F., Balart R. (2017). A combination of three surface modifiers for the optimal generation and application of natural hybrid nanopigments in a biodegradable resin. J. Mater. Sci..

[38] Bustamante J., van Stempvoort S., García-Gallarreta M., Houghton J.A., Briers H.K., Budarin V.L., Matharu A.S., Clark J.H. (2016). Microwave assisted hydro-distillation of essential oils from wet citrus peel waste. J. Clean. Prod..

[39] Bascialla G., Regazzoni A.E. (2008). Immobilization of anionic dyes by intercalation into hydrotalcite. Colloids Surfaces A Physicochem. Eng. Asp..

[40] Vankar P.S. (2017). Structure-mordant interaction, replacement by biomordants and enzymes. Natural Dyes for Textiles: Sources, Chemistry and Applications.

[41] Nguemtchouin M.G.M., Ngassoum M.B., Chalier P., Kamga R., Ngamo L.S.T., Cretin M. (2013). Ocimum gratissimum essential oil and modified montmorillonite clay, a means of controlling insect pests in stored products. J. Stored Prod. Res..

[42] Noudem J.A., Mbouga M.G.N., Kaptso K.G., Khalfaoui M., Noumi G.B. (2017). Saponins-Clay Modified Materials: A New Approach Against Callosobruchus Subinnotatus In Stored Products. Int. J. Sci. Technol. Res..

[43] Olopade B.K., Oranusi S., Nwinyi O.C., Njobeh P.B., Lawal I.A. (2019). Characterization of Nanoformulations from Montmorillonite clay for the decontamination of zearalenonein cereals using X-ray Diffraction Technique. Proceedings of the Journal of Physics: Conference Series.

[44] Campos-Requena V.H., Rivas B.L., Pérez M.A., Garrido-Miranda K.A., Pereira E.D. (2018). Release of essential oil constituent from thermoplastic starch/layered silicate bionanocomposite film as a potential active packaging material. Eur. Polym. J..

[45] Parvinzadeh Gashti M., Moradian S. (2012). Effect of nanoclay type on dyeability of polyethylene terephthalate/clay nanocomposites. J. Appl. Polym. Sci..

[46] Toshniwal L., Fan Q., Ugbolue S.C. (2007). Dyeable polypropylene fibers via nanotechnology. J. Appl. Polym. Sci..

[47] Razafimahefa L., Chlebicki S., Vroman I., Devaux E. (2005). Effect of nanoclay on the dyeing ability of PA6 nanocomposite fibers. Dye. Pigment..

[48] Conesa A., Manera F.C., Brotons J.M., Fernandez-Zapata J.C., Simón I., Simón-Grao S., Alfosea-Simón M., Martínez Nicolás J.J., Valverde J.M., García-Sanchez F. (2019). Changes in the content of chlorophylls and carotenoids in the rind of Fino 49 lemons during maturation and their relationship with parameters from the CIELAB color space. Sci. Hortic. (Amsterdam)..

[49] Luzi F., Fortunati E., Jiménez A., Puglia D., Pezzolla D., Gigliotti G., Kenny J.M., Chiralt A., Torre L. (2016). Production and characterization of PLA_PBS biodegradable blends reinforced with cellulose nanocrystals extracted from hemp fibres. Ind. Crops Prod..

[50] Mahmoodi A., Ghodrati S., Khorasani M. (2019). High-Strength, Low-Permeable, and Light-Protective Nanocomposite Films Based on a Hybrid Nanopigment and Biodegradable PLA for Food Packaging Applications. ACS Omega.

[51] Marchante V., Benavente V., Marcilla A., Martínez-Verdú F.M., Beltrán M.I. (2013). Ethylene vinyl acetate/nanoclay-based pigment composites: Morphology, rheology, and mechanical, thermal, and colorimetric properties. J. Appl. Polym. Sci..

[52] Xia T., Ye Y., Qin W.L. (2019). Acrylonitrile–butadiene–styrene colored with a nanoclay-based filler: Mechanical, thermal and colorimetric properties. Polym. Bull..

[53] Bala P., Samantaray B.K., Srivastava S.K. (2000). Dehydration transformation in Ca-montmorillonite. Bull. Mater. Sci..

[54] Frost R.L., Martens W.N., Erickson K.L. (2005). Thermal decomposition of the hydrotalcite: Thermogravimetric analysis and hot stage Raman spectroscopic study. J. Therm. Anal. Calorim..

[55] Moujahid E.M., Lahkale R., Ouassif H., Bouragba F.Z., Elhatimi W. (2018). New organic dye/anionic clay hybrid pigments: Preparation, optical properties and structural stability. Dye. Pigment..

[56] Nejadebrahim A., Ebrahimi M., Allonas X., Croutxé-Barghorn C. (2020). Methylene blue-clay nano-pigment as a new photosensitizer for preparing three-component photoinitiating systems with high thermal stability. Dye. Pigment..

[57] Raha S., Quazi N., Ivanov I., Bhattacharya S. (2012). Dye/Clay intercalated nanopigments using commercially available non-ionic dye. Dye. Pigment..

[58] Ramos M., Dominici F., Luzi F., Jimenez A., Garrigós M., Torre L., Puglia D. (2020). Effect of Almond Shell Waste on Physicochemical Properties of Polyester-Based Biocomposites. Polymers (Basel)..

[59] Chiang M.F., Chu M.Z., Wu T.M. (2011). Effect of layered double hydroxides on the thermal degradation behavior of biodegradable poly(l-lactide) nanocomposites. Polym. Degrad. Stab..

[60] Ha J.U., Xanthos M. (2010). Novel modifiers for layered double hydroxides and their effects on the properties of polylactic acid composites. Appl. Clay Sci..

[61] Wei Z., Chen G., Shi Y., Song P., Zhan M., Zhang W. (2012). Isothermal crystallization and mechanical properties of poly(butylene succinate)/layered double hydroxide nanocomposites. J. Polym. Res..

[62] Marchante V., Marcilla A., Benavente V., Martínez-Verdú F.M., Beltrán M.I. (2013). Linear low-density polyethylene colored with a nanoclay-based pigment: Morphology and mechanical, thermal, and colorimetric properties. J. Appl. Polym. Sci..

[63] Tan L.C., He Y., Qu J. (2019). ping Structure and properties of Polylactide/Poly(butylene succinate)/Organically Modified Montmorillonite nanocomposites with high-efficiency intercalation and exfoliation effect manufactured via volume pulsating elongation flow. Polymer (Guildf)..

[64] Tan L., Chen Y., Zhou W., Ye S., Wei J. (2011). Novel approach toward poly(butylene succinate)/single-walled carbon nanotubes nanocomposites with interfacial-induced crystallization behaviors and mechanical strength. Polymer (Guildf)..

[65] Monshizadeh M., Seifi S., Hejazi I., Seyfi J., Khonakdar H.A. (2019). Enhanced properties of poly(lactic acid) by concurrent addition of organo-modified Mg-Al layered double hydroxide (LDH) and triethyl citrate. J. Thermoplast. Compos. Mater..

[66] Katiyar V., Gerds N., Koch C.B., Risbo J., Hansen H.C.B., Plackett D. (2010). Poly l-lactide-layered double hydroxide nanocomposites via in situ polymerization of l-lactide. Polym. Degrad. Stab..

[67] Schadler L.S. (2003). Polymer-Based and Polymer-Filled Nanocomposites. Nanocomposite Science and Technology.

[68] Ollier R., Rodriguez E., Alvarez V. (2013). Unsaturated polyester/bentonite nanocomposites: Influence of clay modification on final performance. Composit. Part A Appl. Sci. Manuf..

[69] Qin Y., Li W., Liu D., Yuan M., Li L. (2017). Development of active packaging film made from poly (lactic acid) incorporated essential oil. Prog. Org. Coat..

[70] Othman S.H., Ling H.N., Talib R.A., Naim M.N., Risyon N.P., Saifullah M. (2019). PLA/MMT and PLA/Halloysite Bio-Nanocomposite Films: Mechanical, Barrier, and Transparency. J. Nano Res..

